# Ambulatory health services utilization in patients with dementia - Is there an urban-rural difference?

**DOI:** 10.1186/1476-072X-9-59

**Published:** 2010-11-17

**Authors:** Daniela Koller, Marion Eisele, Hanna Kaduszkiewicz, Gerhard Schön, Susanne Steinmann, Birgitt Wiese, Gerd Glaeske, Hendrik van den Bussche

**Affiliations:** 1 University of Bremen, Centre for Social Policy Research, Division Health Economics, Health Policy and Outcomes Research, Parkallee 39, 28209 Bremen, Germany; 2 Department of Primary Medical Care, Center for Psychosocial Medicine, University Medical Center Hamburg-Eppendorf, Martinistr. 52, 20246 Hamburg, Germany; 3 Department of Medical Biometry and Epidemiology, Center for Experimental Medicine, University Medical Center Hamburg-Eppendorf, Martinistr. 52, 20246 Hamburg, Germany; 4 Centre for Biometry, Medical Informatics and Medical Technology, Institute for Biometry, Hannover Medical School, OE 8410, 30625 Hannover, Germany

## Abstract

**Background:**

Due to demographic changes and an un-equal distribution of physicians, regional analyses of service utilization of elderly patients are crucial, especially for diseases with an impact like dementia. This paper focuses on dementia patients. The aim of the study is to identify differences in service utilization of incident dementia patients in urban and rural areas.

**Methods:**

Basis for the analysis were all insured persons of a German Health Insurance fund (the GEK) aged 65 years and older living in rural and urban areas. We focussed on physician contacts in the outpatient sector during the first year after an incidence diagnosis of dementia. Special attention was given to contacts with primary care physicians and neurologists/psychiatrists. The dementia cohort was analyzed together with a non-dementia control group drawn according to age, gender and amount of physician contacts. Uni- and bivariate as well as multivariate analysis were performed to estimate the influences on service utilization.

**Results:**

Results show that the provision of primary care seems to be equally given in urban and rural areas. For specialists contacts however, rural patients are less likely to consult neurologists or psychiatrists. This trend can already be seen before the incident diagnosis of dementia. All consultations rise in the quarter of the incident dementia diagnosis compared to the control group. The results were also tested in a linear and a logistic regression, showing a higher chance for persons living in urban areas to visit a specialist and an overall higher rate in service utilization for dementia patients.

**Conclusions:**

Because of a probable increase in the number of dementia patients, service provision has to be accessible even in rural areas. Due to this and the fact that demographic change is happening at different paces in different regions, regional variations have to be considered to ensure the future service provision.

## Background

Accessibility to health services, especially for persons with a disease with such an impact as dementia, is important in order to ensure guideline-orientated diagnosis and optimal therapy.

Dementia is a disease with a major impact on patients, relatives and society; and its importance will grow in the future due to demographic changes. In Germany, the population aged 65 years and older consisted of over 15 million persons in the year 2005 (19% of the total population). Based on the statistic assumptions, this number will increase to between 21 and 25 million persons (over 30% of the total population) in the year 2050 [1]. Optimal health care at the onset of the disease is needed to ensure a higher quality of life and a longer independent living with dementia. It is also crucial for the community, since caring for patients is an important economic factor. Direct costs for dementia range between 9.000 and 16.0000 Euros in European countries per year [2].

### Access to ambulatory health care

Access to physicians is a widely discussed topic. Important barriers to access primary care in Europe were analyzed by Schoen et al., who identified costs for medication and waiting times to see a specialists as well as the time a physician spends with a patients as major influencing factors [3]. Also, the physical ability to access health care is important, i.e. public transportation or the availability of physicians within walking distance. There is a rather controversial discussion on the optimal number of general practitioners or specialists per inhabitants in an area. Internationally, the scientific literature on service provision in rural areas points to a lack of physicians and to possible incentives to make the work in rural areas more attractive for physicians (e.g. for America, see [4]; for Scotland, see [5]), while clear indicators to measure over- or underprovision of physicians or quality of care are lacking in Germany [6,7]. Nevertheless, it can be assumed that access especially to specialists is more difficult in rural areas than in urban areas due to a lower physician density and less public transport.

In Germany, the physician atlas states that general practitioners are found throughout most areas. Regionally, Germany can be subcategorized in federal states (Länder), those in administrative districts (Bezirke), those into counties (Kreise) and those into municipalities (Gemeinden). Counties often include a county seat and a surrounding rural area. On a county level, the service supply is sufficiently given; only in some areas in the north-east an under-supply is noticeable on county level. However, it has to be considered that counties are rather large areas combining more urbanized and more rurally influenced regions with differences in population density and infrastructure. Therefore, it cannot be assumed that the density of general practitioners is equally distributed in a county [8].

The situation regarding specialist care is different. In this publication we focus on neurologists and psychiatrists. While, according o the physician atlas, in most areas there seems to be an oversupply on a county-level, there is region in the federal state Saxony-Anhalt that has an undersupply regarding neurologists and psychiatrists (NPs) [8]. For specialists even more than for the group of GPs, regional variance within a county is probable because of different population densities within the counties. The stated oversupply on a county-level therefore needs to be addressed critically. Hence, we will differentiate on a smaller level, the municipalities.

### Regional differences in utilization

Differences in service utilization for persons living in urban or rural areas have been described, even though studies concerning health care utilization by elderly patients in rural areas, especially by dementia patients, are scarce. One way to determine poor service provision or utilization is to analyze hospital admissions for ambulatory care sensitive conditions (ACSC). The hypothesis is that those admissions could be prevented in a sufficient ambulatory care system. Mobley et al. analyzed all ACSCs in the US, showing that if the degree of poverty for elderly is high in rural areas, those persons tend to have a higher admission rate for ACSCs than their urban counterparts [9]. Laditka showed that persons in low supply areas had higher risks for ambulatory care sensitive hospitalization. Areas with adequate supply on the other hand showed a lower risk [10].

That service supply is not only a question of the crude distance but also a question of social space is discussed in Castleden et al. study on geographic differences in rural palliative care, identifying a complex combination of distance, location, aesthetics and sites of care in Canada. The authors conclude that the growing elderly population in rural areas leads to difficulties in formal and informal service provision at the end of live, since the modes of transportation and condition of transportation possibilities can differ widely [11].

There is few research published focussing especially on service utilization by patients with dementia. Morgan et al. describe a rural and remote memory clinic for Canada, after reviewing existing studies showing limited access availability for services, problems concerning transportations and distances [12]. In another study on dementia patients in Canada, the authors conclude that while there are no differences in reported unmet health needs, rural dementia patients reported that needed care was unavailable or not accessible [13]. In a study from Germany, Donath et al. analyzed diagnostic procedures and dementia therapy in rural and urban areas, finding only differences in the rate of imaging techniques as diagnostic instruments. Differences concerning other procedures such as referrals to specialists or physical examinations were not found [14].

Especially for elderly and patients with dementia, regional differences in nursing care availability are also of importance. Rothgang et al. found various differences in the supply of nursing facilities (ambulatory as well as stationary) between federal states and rural and urban areas in Germany [15]. This closer regional differentiation will grow in importance since the demographic change occurs unbalanced between the federal states in Germany. By the year 2050, the portion of persons aged 65 years and older will range between 54% (in Bremen) and 87% (in Thüringen) [16,17].

### Defining rural and urban

When evaluating health behaviour in rural areas, one faces the problem of defining "urban" and "rural". While this is already a difficult task for one specific region or country, it is hardly possible to find a common international standard; i.e. a rural area in Germany has to be discussed differently than rural areas in larger countries like the USA, Canada or Australia.

Castleden et al. describe in their introduction the geographic point of view, identifying rurality as a socially and culturally constructed phenomenon, and list a number of studies focus on inequalities in services provision, health behaviour and rural lifestyles [11,18].

Other than the theoretical side of "urban" and "rural", it is crucial to find an empirical definition for analysis. Various factors can be considered, such as population density, absolute number of inhabitants, distance to nearest agglomerated area, infrastructure of transportation, etc. This problem has been pointed out before [19].

In this paper, we use the municipality types defined by the Federal Institute for Research on Building, Urban Affairs and Spatial Development (BBR) (see methods section).

### German Health Insurance System

To interpret the utilization patterns of the patients, information on the German health insurance system is necessary. In Germany, next to the hospital sector there is an independent outpatient/ambulatory system. Physicians in the hospital sector are not working in ambulatory care and vice versa. The ambulatory sector consists of physicians working alone or in group offices. Usually, the primary care physician is the person first contacted by patients, but he does not have a gate-keeper function, all patients can contact physicians of any specialization at any time. A fee of 10 Euros has to be paid for each quarter for ambulatory care; to avoid paying this fee at each consultation, a referral is needed.

### Aim of the study

Based on this background, this study aims to evaluate the actual ambulatory medical services utilization of dementia patients in the German Statutory Health Insurance in the year before and after the diagnosis of dementia in rural and urban areas. Since studies in this area are scarce, especially in Germany, our study tries to fill this gap. We assume that the service utilization, mainly for specialists care, is lower in rural areas. An overall analysis of the service utilization with no geographic focus has already been made with these data [20].

## Methods

The analyses are based on the population of the GEK, a German nationwide health insurance fond insuring about 1.7 million people (in 2007). We selected all persons who were at least 65 years old and were continuously insured in the GEK from 2004 to 2006. To ensure incident cases of dementia, a person must not have a diagnosis of dementia in at least all four quarters of 2004 and must have three diagnoses in following four consecutive quarters. The observational period covers the incidence quarter and the year before and after. To ensure that a consultation was made due to the dementia diagnosis, a control group was formed with a 4:1 matching with the criteria age, sex, and number of contacts with physicians four quarters before the Q5 (incidence quarter for the dementia patients). The selection of patients and controls has been described more extensively elsewhere [20].

To identify possible rural and urban differences in service utilization, therapy, morbidity or mortality we created a regional variable. Due to data protection policies, the current living address of the insured persons is not part of the data. We therefore used the "Siedlungsstrukturellen Gemeindetypen" (municipality types based on the settlement system classified by the Federal Institute for Research on Building, Urban Affairs and Spatial Development, "BBR"). Through datasets offered by the German Post office and the Federal Statistical Office we created an assignment table that enables to match postal codes with the municipality types. This data was processed by the GEK to maintain the anonymity of each person so that eventually every person was categorized into a municipality type (1 through 17; 1 being bigger core cities and 17 being rural municipalities of low density). The 17 categories are sub-categorized into the three groups "agglomerated area", "urbanized area" and "rural areas, though an ordinal scale is not given. We re-classified them into rural and urban; the categories 1 through 7, 9 and 10 were classified as being urban, 8 and 11 through 17 were classified as being rural.

To determine the utilization of health services, we included all contacts the persons had in ambulatory care in the year before and after the first diagnosis of dementia for the dementia group and the corresponding 8 quarters for the control group, respectively. We analyzed visits to a) all physicians, b) primary care physicians (PCP) and c) neurologists and psychiatrists ("neuropsychiatrists", NP). A contact is defined as a consultation of one physician, if that specific physician was contacted more than once during the quarter, every visit was counted. To evaluate how many different physicians were contacted, we also included the number of contacted physicians in our analysis. To analyze the effects on health care utilization, we also adjusted for other individual characteristics in multivariate linear and logistic models.

Local ethical committees approved the study. All statistical analyses were performed with SAS (Version 9.2) and R.

## Results

Table 1 shows the basic characteristics of the dementia cohort. Dementia patients in urban and rural environments do not differ according to age and sex, even though there are slightly more people of the youngest age group living in an urban environment. There is also no noticeable difference according to the percentage of persons with a nursing care dependency (which is classified into three levels in the German health and nursing care system). The missing values are due to the not possible classification into the urban or rural group and are therefore not part of the following analysis.

**Table 1:** Basic characteristics of dementia patients and control group

	Dementia group		Control group	
	***Urban***	***Rural***	***Urban***	***Rural***

*N*	1329	517	5249	2121
Missing	2		22	
*age (mean; SD)*	78.75 (7.44)	78.62 (7.27)	78.81 (7.36)	78.57 (7.36)
*65-74 years (%)*	30.7	30.56	30.25	31.26
*75-84 years (%)*	47.03	47	47.49	46.86
*85 years and older (%)*	22.27	22.44	22.25	21.88
*Nursing care dependency (yes)*	35.52	34.62	10.4	11.5
*Sex (female)*	47.93	46.62	47.4	48

**Figure 1:**
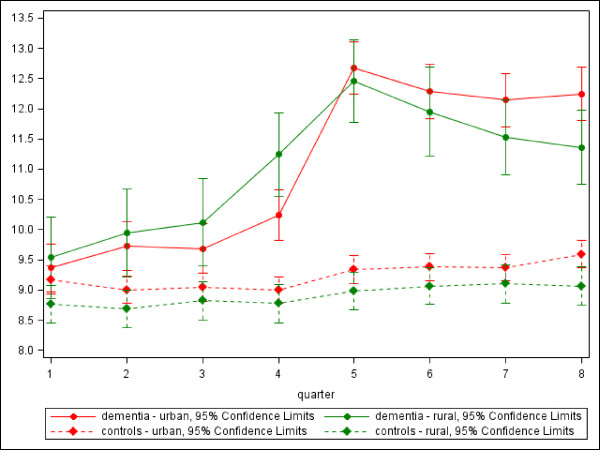
**Amount of physician contacts (mean) for dementia patients and controls in the years before and after the incident diagnosis**.

Figure 1 shows the amount of physician contacts, regardless of physician specialization, of the dementia group and the control group in rural and urban setting.

No significant differences were found between patients with dementia in rural or urban setting, even though the differences between the two groups grow until the 8^th ^quarter (approx. one year after diagnosis). Without reaching the level of statistical significance, this tendency demonstrates that persons living in and rural environment could not consult physicians as often as patients in urban areas. This effect is not observable for the control group.

**Figure 2:**
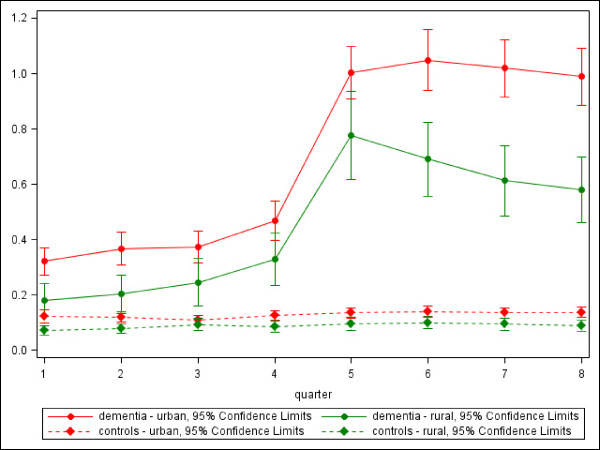
**Mean number of contacts with NP for dementia patients and controls in the years before and after the incident diagnosis**.

Subsequently, we analyzed the utilization in a more detailed way; focussing on the number of contacts with PCPs and NPs (Figure 2).

While the same rise in consultations of PCPs by dementia patients in Q5 and before is visible similar to the amount of physician contacts, there is no significant difference between the urban and the rural population. In fact, persons living in a rural environment seem to visit a PCP a little bit more frequently than their urban counterparts. A very different tendency can be found concerning the visits to NPs (Figure 2). There too, an obvious increase in contacts is observable in the dementia group, but almost throughout the whole observation period patients with dementia in urban areas contact significantly more often a NP than those living in rural areas. Here, two other important facts have to be pointed out: First, the patients within the incidence group visit NPs more often before their incident diagnosis of dementia than the controls in the same time period, leading to the assumption that cognitive deficits might already be apparent before the diagnosis is eventually coded by the physician. The second point is that even though urban dementia patients contact NPs significantly more often than rural patients, the group of dementia patients who ever contact an NP throughout the eight quarters is 50% only. Of all dementia patients living in urban areas, 52.8% have at least one contact to an NP, those living in rural areas 42.8%, respectively (p for chi-square: < .0001).

In a next step we further analyzed how many different physicians were contacted by the insured persons (not shown as a figure). In this analysis, it does not matter how often one specific physician was seen during the quarter. These physicians again include all PCPs as well as all specialists, serving as an indicator of the persons overall health care utilization; i.e. if regular general check-ups, eye-exams, orthopaedic or urologic/gynaecologic visitations are made. Here the familiar increase in Q5 in the dementia group is also noticeable. The people living in urban areas visit more different physicians than people living in rural environments. In the dementia group, this difference is strongly visible in the quarters after diagnosis; however, it is not statistically significant. For the control group, significant differences between urban and rural persons are noticeable throughout the whole observational period.

### Regressions

To control for individual factors, we performed multivariate regressions. For the number of physician contacts a linear regression (response variable: number of contacts) and the consultation of a NP a logistic regression (response variable: person visited a NP: yes/no). The control variables in both regressions were: age, sex, nursing care dependency, community setting and dementia yes/no. Both regressions refer to the Q5 and the Q7. In the Q5, the service utilization differed a lot from the quarters before, so we chose to analyze the Q7 which gives information on service utilizations after the "impact" of the first diagnosis. The Q7 therefore shows a more "regular" service utilization for the patients with dementia already visible in the graphs shown before. Table 2 illustrates the results of the linear regression.

**Table 2:** Linear Regression - Influences on the number of physician contacts

Number of physician contacts						
	**Incidence Quarter (Q5)**			**half year after incidence (Q7)**		

***Variable***	***Param.*** ***Est.***	***95% Confidence*** ***Limits***	***p (t)***	***Param.*** ***Est.***	***95% Confidence*** ***Limits***	***p (t)***


sex (female)	-0.42	-0.75 - -0.08	0.0158	-0.38	-0.7 - -0.05	0.0253
age	-0.04	-0.07 - -0.02	0.0004	-0.05	0.07 - -0.02	0.0001
Nursing care dependency (yes)	3.69	3.21 - 4.17	<.0001	3.91	3.44 - 4.38	<.0001
Community setting (rural)	-0.36	-0.72 - 0.00	0.0509	-0.38	-0.73 - -0.03	0.0335
Dementia/control (dementia)	2.49	2.08 - 2.91	<.0001	1.72	1.32 - 2.13	<.0001

It can be seen that the major influences on the number of consultations are nursing care dependency, being responsible for 3.7 additional visits within the incidence quarter, and the diagnosis of dementia with 2.5 additional visits, respectively. All other parameters show statistical significance due to the high number of persons in the sample, but do not show a major influence on the number of consultations. The results do not differ between the diagnosis quarter and half a year after diagnosis in most variables. The only factor that is not significant in the incidence quarter but half a year after is the community setting, although compared to the values of dementia and nursing care dependency, the estimate is rather low.

Table 3 shows the results of the logistic regression.

**Table 3:** Logistic Regression - Influences on chance to visit an NP

Chance to visit a NP						
	**Incidence Quarter (Q5)**			**half year after incidence (Q7)**		

**Variable**	**Odds** **Ratio**	**95% Confidence** **Limits**	**p (chisq)**	**Odds** **Ratio**	**95% Confidence** **Limits**	**p (chisq)**

sex (female)	1.06	0.92 - 1.23	0.42	0.99	0.85 - 1.14	0.84
age	0.95	0.94 - 0.96	<.0001	0.95	0.94 - 0.96	<.0001
Nursing care dependency (yes)	1.02	0.84 - 1.23	0.87	1.3	1.08 - 1.57	0.01
Community setting (rural)	0.71	0.61 - 0.84	<.0001	0.71	0.6 - 0.83	<.0001
Dementia group	8.41	7.26 - 9.74	<.0001	7.77	6.69 - 9.04	<.0001

The largest influence on a NP consultation is the dementia diagnosis in the quarter of the first diagnosis. Those patients have a 8.4fold chance to visit a NP compared to the control group. While age reaches the level of statistical significance, it does not influence the NP visit noticeably, overall the younger patients have a greater chance to visit a NP. Other than in the linear regression on all physician contacts, the regional variable is important for the NP visits even under the control of other factors. Persons living in urban surroundings have a significantly higher chance (of about 43%) to visit an NP compared with their rural counterparts in the incidence quarter this chance persists over the following quarters.

## Discussion

Our analysis identified differences in utilization of ambulatory medical services between urban and rural dementia patients. While urban patients visit more NPs in the year before and after the first dementia diagnosis, rural patients tend to contact their PCP more often but less NPs. Even after controlling for age, sex and the existence of nursing care dependency, the chance to visit an NP is much lower in the rural population than in urban persons. These finding are not in line with the results of Donath et al., who did not find major visit frequency differences between rural and urban dementia patients besides the use of imaging techniques of the diagnosis, which were more often applied for urban dementia patients. They analyzed the referrals to specialists, and did not find any differences between the urban and rural population [14]. An explanation might be the different data background. While our analysis is based on claims data, the Donath study is based on primary data through participating GPs, so differences could occur through methodological differences and therefore should be tested in future studies.

The lower utilization in rural areas can have various reasons we can only speculate about. Several factors have been identified in the literature. First of all, the distance to the service provider can be far and the transportation difficult for patients as for carers [21]. Individual transport can depend on a caregiver who has the time and resources. As the number of single households grow and children more often do not live close to their parents, this is an issue of growing importance. Possible public transport and help for elderly patients, especially with mental disorders, has to become a focus in public discussions.

Another reason for different service utilization can be a different lifestyle and beliefs in rural areas, including a stronger sense of community which could result in more personal assistance and less professional consultations. Also, social stigmatization of mental diseases has to be taken into account. This might lead to less service utilization because a patient might not want to address mental problems. It can also lead to a later diagnosis or no diagnosis at all, since physicians might not be comfortable to address existing problems [18,21]. Still, the differences we found are not consistently significant throughout the total observational period. Only the number of contacts with NPs is already significantly different from urban and rural dementia patients before their incidence. Concerning the number of contacted physicians, these differences can be seen as a tendency for the quarters from the incident diagnoses on. If this points to a lack of service provision or if the patients are treated adequately by fewer physicians needs to be addressed in future research.

Remarkably, half of the incident cases with contact to a NP had several contacts in the year after the incidence quarter (van den Bussche H, Eisele M, Koller D, Wiese B, Kaduszkiewicz H, Glaeske G, Steinmann S, Wegscheider K, Schön G: Specialist involvement and referral patterns in ambulatory medical care for patients with dementia in Germany - Results of a case-control-study. Submitted.). For these continuous utilizers, the NP is obviously taking over permanent care and co-treatment function, a phenomenon not found in the control sample. These continuous utilizers are more often urban inhabitants. Only 57.3% of all rural patients visiting an NP in the quarter of incidence still visit a NP four quarters later (compared to 72.9% in the urban sample). This can partly explain the growing decrease in utilization between urban and rural regions as seen in Figure 2.

### Strengths and limitations

Our study identified differences in urban and rural service utilization in dementia patients in specialist consultations, not for primary care. It is based on a secondary data analysis of health insurance claims data and therefore has some limitations. Due to data validity, we included only dementia patients insured over the whole study period which might lead to the exclusion of some patients, especially those who died within the year after the first diagnosis of dementia. Also, the regional differentiation of residence is only possible according to rural and urban, an exact position of the patient on the map is not possible because of data privacy. An analysis of variations between different urban or different rural areas is therefore not possible. Also, the data is collected for financial purposes and therefore does not include more variables on the socio-economic background of the insured persons. I.e., some studies in the US found racial differences in service provision [10,22]. Yet, due to the large database we were able to identify real service utilization differences and can generate new research questions. Compared to field studies, we could also include dementia patients who are institutionalized, the very old as well as patients with other disabilities usually not reachable for scientific research.

## Conclusions

Regional variations between urban and rural persons with dementia do exist, especially concerning specialists treatment after the incident diagnosis of dementia. In further research, the focus should lie on the consequences - it has to be addressed if less resource utilization leads to a lack of treatment and a worse course of disease or if the PCPs in rural areas compensate the lack of specialists. So the question if more service also leads to more health needs to be addressed. Another question would be the closer analysis of the influence of the socioeconomic and cultural background on service utilization. All this could help health services research, local policy makers and communities to inform about and help accessing an optimal health care for dementia patients.

## Competing interests

GG received funding to analyse data of several health insurance companies, for instance the GEK. All other authors declare that they have no competing interest.

## Authors' contributions

DK performed statistical analysis and wrote the main body of the text; ME contributed to the study design and revised the article, HK contributed to the interpretation, GS performed statistical analysis and revised the paper, SS contributed in data management and statistical analysis, BW contributed in the conception of the cohort and statistical analysis, GG was responsible for the acquisition of data and study conception and HvdB contributed to study design, writing text and interpretation. All authors have read and approved the final manuscript.
